# Interviewer effects on underreporting of traditional contraceptive use: evidence from National Family Health Survey-5

**DOI:** 10.3389/fdata.2026.1812391

**Published:** 2026-08-20

**Authors:** Rupalee Singh Chauhan, Laxmi Kant Dwivedi, Priyanka Dixit, Shiva S. Halli

**Affiliations:** 1 Department of Survey Research and Data Analytics, International Institute for Population Sciences, Mumbai, India; 2 School of Health Systems Studies, Tata Institute for Social Sciences, Mumbai, India; 3 Rady Faculty of Health Sciences, College of Community and Global Health, University of Manitoba, Winnipeg, MB, Canada

**Keywords:** cross-classified multilevel model, India, interviewers' characteristics, National Family Health Survey-5, traditional contraceptive

## Abstract

**Background:**

Socioeconomic alignment between interviewers and respondents may play a critical role in shaping the accuracy of reported reproductive histories. This study investigates how interviewer characteristics contribute to the reporting of adoption of traditional contraceptive methods using reproductive event calendar data from the fifth round of the National Family Health Survey (2019–21) in India.

**Data and methods:**

Using contraceptive calendar data, a cross-classified multilevel model was employed to assess the interviewer's influence on the reporting of traditional contraceptive method use. The analysis included women aged 15–49 years who reported contraceptive use in the calendar month(s), and the interviewers interviewed them.

**Results:**

Multilevel cross-classified models show that interviewer characteristics significantly influence reporting of traditional contraceptive use following periods of non-use, childbirth, or pregnancy termination. Women interviewed by older interviewers (≥30 years; adjusted odds ratio (AOR) = 1.40, *p* < 0.01) and those who shared the same religion (AOR = 1.21, *p* ≤ 0.01) and marital status (AOR = 1.42, *p* < 0.01) as the interviewer were more likely to report traditional contraceptive method use. While women of the same age group (AOR = 0.89, *p* < 0.05), same-state residence (AOR = 0.82, *p* < 0.01), and same caste (AOR = 0.94, *p* < 0.01) as the interviewer were less likely to report traditional contraceptive method use. Older interviewers (AOR = 0.86, *p* < 0.01), those with secondary education (AOR = 0.54, *p* < 0.01), those belonging to the Muslim religion (AOR = 0.43, *p* < 0.01), and those with a lack of prior experience in conducting surveys (AOR = 0.90, *p* < 0.10) were associated with lower reporting. Moderate (AOR = 1.35, *p* < 0.01) and high (AOR = 1.22, *p* < 0.05) levels of prior experience with the same survey schedule in conducting the survey significantly improved reporting. Distribution of variance across different levels indicates substantial variation at the interviewer level (28.50%), followed by the district (18.50%) and PSU (13.46%) levels, suggesting that interviewer effects play a significant role in shaping the outcome.

**Discussion and conclusion:**

Interviewer characteristics such as education, age, and prior survey experience influence the reporting of traditional contraceptive use. Similarities or differences between interviewers and respondents in characteristics such as age, marital status, religion, caste, and state of residence also affect the accuracy and completeness of reporting traditional contraceptive use in contraceptive calendar data.

## 1 Background

As of 2021–2023, approximately 65% of in-union women worldwide use some form of contraception, with approximately 59% using modern contraceptive methods (United Nations Population Division, 2024). Globally, approximately 92 million women use traditional contraceptive methods, representing approximately 9%−10% of all contraceptive users. The use of traditional methods tends to be associated with cultural preferences, limited access to modern methods, or concerns about side effects of modern contraception (United Nations Department of Economic and Social Affairs, Population Division, 2022). The overall contraceptive prevalence rate among married women aged 15–49 in India increased from approximately 54% (NFHS-4) to approximately 67% (NFHS-5), reflecting substantial growth in contraceptive use. The modern contraceptive prevalence rate is approximately 56.5% as per NFHS-5, with an estimated 159 million women using modern contraception in 2024. The use of traditional methods has doubled as per the last two rounds of the NFHS survey, increasing from 5.7% in NFHS-4, 2015–2016, to 10.2% in NFHS-5, 2019–2021, along with that 21 states in India show an increase in the use of traditional methods of contraceptives (Prakash et al., 2022). Evidence suggested that in Uttar Pradesh, the use of traditional methods increased by more than 20% over the inter-survey period, a rise considerably higher than national averages (Namasivayam et al., 2023), and this increase is not necessarily due to lack of availability and accessibility, but increasing preferences for traditional methods despite government efforts to make more modern spacing methods available for women (Prakash et al., 2022; Namasivayam et al., 2023). Similarly, in West Bengal and Assam, traditional method use has been reported at levels as high as 17%−18%, and nationally, these traditional methods account for approximately one-third (35%) of spacing contraceptive use (Ram et al., 2014).

The detailed use of contraception in large-scale surveys for women in India is mentioned by NFHS data regularly, using the calendar data method.^1^ Despite the widespread and increasing use of contraception, evidence suggests that accurate reporting of contraceptive use, especially for traditional methods, remains a significant challenge in developing countries. In India, traditional contraceptive methods may be particularly prone to underreporting because respondents may not consistently interpret what counts as current use, and reporting in large surveys may be affected by recall and survey-design related errors (Kumar et al., 2022). In developing countries, contraceptive use is often not properly reported, especially for traditional methods, and this happens more often when these methods fail (Polis et al., 2016). It has also been noticed that the highest consistency of contraceptive use reporting was observed among women using sterilization and oral contraceptives (Callahan and Becker, 2012). Past studies have also highlighted that the accuracy of the data collected may be influenced by the interviewer's characteristics, which include their behavior, attitude, and communication style (Cannell et al., 1968). Interviewer bias can affect the respondents' responses, and it will ultimately distort the survey results (Kühne, 2023; Groves, 1989). It is also evident from the research that both the interviewer and respondent play vital roles in the quality of data.

Evidence from existing literature indicates that reporting of contraceptive use varies substantially by method type, with traditional methods being consistently more underreported than modern methods such as sterilization, particularly in reproductive event calendar data (Polis et al., 2016; Rossier et al., 2014). But traditional methods are, and will continue to be, used by women because they are deeply embedded in many cultures and continue to play an important role in pregnancy prevention for millions of women globally (Bertrand et al., 2022). While calendar data are widely used to estimate contraceptive use dynamics (Elkasabi and Assaf, 2021), concerns remain regarding their accuracy, especially for methods that are episodic, culturally sensitive, or socially less acceptable. Parallel research has highlighted that data quality in large-scale surveys is not only influenced by the method of data collection but also by interviewer-related factors, including their socioeconomic background, behavior, communication style, and ability to build rapport with respondents (Cannell et al., 1968; Groves, 1989; Glasner and Van Der Vaart, 2009). Drawing on these two streams of research, this study conceptualizes that greater socioeconomic alignment between interviewers and respondents, such as similarities in marital status, parenthood, place of residence, or shared reproductive experiences, may foster trust and engagement during interviews, thereby improving recall and willingness to disclose reproductive information. Such alignment may be particularly important for reporting traditional contraceptive use, which is more prone to omission and misreporting, particularly in DHS surveys (Rossier et al., 2014; Marston et al., 2017; Rabiu and Rufa'i, 2018; Mairiga et al., 2012). Using reproductive event calendar data from the fifth round of the National Family Health Survey (2022) in India, this study examines how interviewer characteristics contribute to the reporting of traditional contraceptive methods relative to modern methods following non-use of contraception, childbirth, and pregnancy termination, which represent important reproductive transition points at which women are likely to initiate or resume contraception. Building on evidence that later rounds of NFHS have shown improved consistency compared to earlier rounds (Kumar et al., 2022), the present analysis focuses on assessing the quality of NFHS-5 calendar data by investigating the extent to which interviewer characteristics influence reporting accuracy. The overarching objective is to evaluate whether interviewer background plays a critical role in shaping respondents' reporting of traditional contraceptive methods in calendar-based survey data.

## 2 Methods and materials

### 2.1 Data sources

For analysis purposes, the National Family Health Survey (NFHS), a periodic and pivotal data set and a nationally representative cross-sectional study overseen by the Ministry of Health and Family Welfare (MoHFW), Government of India, has been used. NFHS meticulously collects data on various dimensions of demographic, health, and socio-economic indicators such as fertility, maternal and child health, reproductive health, infant and child mortality, including contraceptive use, across all states and union territories at the district level in India. NFHS data in India uses a methodology for collecting data on contraceptive use in calendar form, which entails the systematic documentation of contraceptive use over a 60-month time period from the date of interview with the respondent. It provides a detailed and nuanced understanding of reproductive behaviors and reproductive practices (see text footnote 1).

For this study, analysis was conducted on the fifth round of the survey by utilizing the Fieldworker Characteristics file (FW file) and Individual file (IR file). The individual file contains data on women respondents aged 15–49 years, who were interviewed by trained female interviewers in accordance with NFHS survey protocols. The International Institute for Population Sciences (IIPS), which serves as the lead agency for the survey, collaborated with 17 field organizations to conduct data collection. The survey collected detailed information from 636,699 households, involving 724,115 women across 707 districts nationwide during the period 2019–2021, achieving an individual response rate of 97%. It also included information about the interviewer who collected the survey data. A two-stage sample design was used with probability proportional to size to select the primary sampling units (PSUs) at the first stage, where villages served as primary sampling units (PSUs) for rural areas, and census enumeration blocks (CEBs) were selected from urban areas. In the second stage, a fixed number of households (22) were systematically sampled from the selected PSUs (see text footnote 1). The sampling and data collection procedure is well documented on the DHS program web portal (see text footnote 1). Out of 7,127, duplicate fieldworker entries from 2010 were removed, and ultimately only 2,950 female interviewers were retained in the final dataset for the analysis. The FW file contains information about interviewer characteristics collected via a self-administered questionnaire at the outset of fieldwork. Upon completion of training, each interviewer was assigned a unique numeric identification code (ID), which facilitated linking fieldworker data with respondent data. During data cleaning for the study purpose, duplicate interviewer IDs were identified because, in some cases, replacement interviewers were assigned the same ID when the original interviewer left the survey. These duplicate entries were removed to ensure a one-to-one linkage between interviewer and respondent data.

### 2.2 Outcome variable

The contraceptive calendar recorded month-by-month information in a string format on all reproductive events for the last 5 years prior to the survey (60 months). From this contraceptive calendar information, the outcome variable is generated by categorizing the women into two groups: women who did not report opting for a traditional contraceptive method following periods of non-use of contraception, childbirth or pregnancy termination, and those women who reported opting for a traditional contraceptive method after following periods of non-use of contraception, childbirth, or pregnancy termination. Female sterilization, male sterilization, emergency contraception, and missing were dropped from the study, as previous studies have reported high consistency in sterilization reporting (Callahan and Becker, 2012).

### 2.3 Explanatory variables

In this study, we have examined various demographic characteristics of interviewers to understand their potential influence, primarily on contraceptive calendar data. These included age in completed years categorized into four groups (15–20, 21–25, 26–30, 30 and above) as well as interviewer age relative to women respondent coded as younger than, same as, older than the respondent, education level also used in two groups (primary, secondary, higher) and interviewer education also categorized as relative to women respondent (same and different), religion (Hindu, Muslim, other), religion also used as same or different relative to women respondent, caste (ST, SC, OBC, other), caste also used as same or different relative to women respondent, previous work experience in NFHS/ Demographic Health Survey (DHS), and any other surveys along with variable as employment by agency (yes, no), marital status (ever married, never married), children ever born (no children, have children) and place of residence (rural, urban). Along with that, another variable was created to account for the total number of interviews done by the interviewer in the survey period (low with a mean of 16.6 interviews, medium with a mean of 28.4 interviews, and high with a mean of 41.3 interviews). These analyses aim to uncover any potential biases or influences interviewer characteristics may have on survey responses, particularly concerning sensitive topics such as family planning methods.

### 2.4 Control variables

To ensure a comprehensive examination, the study also adjusted for several potential confounders. As the study was about contraceptive use pattern and reporting, it was imperative to include variables relevant to women: age of women respondents in completed years (15–20, 21–25, 26–30, 30 and above), current marital status (never married, ever married), wealth quintile (poorest, poor, middle, rich, richest), place of residence (rural, urban), caste of respondents (Scheduled Tribes/Scheduled Castes, Other Backward Classes, [including None of them and Don't know]), religion (categorized as Hindu, Muslim, [including Christians, Sikhs, Buddhists/Neo-Buddhists, Jains, Jews, Parsis/Zoroastrians, individuals reporting no religion, and other religions]). By including the above variables as control variables in this study, the study aimed to diminish the confounding effect and enhance the research findings.

### 2.5 Methodology

#### 2.5.1 Statistical analysis

Analysis was performed using STATA 16 and MLwiN Version 3.05. Descriptive statistics, coupled with bivariate analysis, were used to explore the distribution of interviewer characteristics. The socioeconomic and demographic profiles of interviewers were presented using descriptive analysis. Non-response among women was adjusted by applying appropriate sampling weights during analysis. Moreover, a multilevel cross-classified model analysis was utilized to address the objectives of the study. The findings were conveyed through adjusted odds ratios (AORs), accompanied by a 95% confidence interval (CI). Furthermore, the intra-class correlation coefficient (ICC) was calculated from a cross-classified random intercept multivariable four-level logit model, which was used to assess the effect of interviewers on the above-defined outcome variables while adjusting for all the independent variables mentioned above.

#### 2.5.2 Cross-classified multilevel model

A four-level, cross-classified, random intercept multivariable logit model was used to examine interviewer effect on the outcome variables. The result for the ith respondent—interviewed by the jth interviewer at the kth PSU in the lth district—was specified in this model. Each interviewer worked for numerous PSUs and districts, and each PSU and district used multiple interviewers, resulting in a cross-classification of the random intercept model. Unlike classic PSU-district models, this system does not have a perfectly hierarchical structure. To disentangle the micro-macro interactions when the contextual level does not present a clear or “natural” structure, we used a cross-classified model. This approach allows for more flexible specifications, making it a valuable tool for analyzing complex, non-hierarchical data relationships (Zaccarin and Rivellini, 2002). The following flowchart shows the hierarchical structure.

Socioeconomic and demographic factors at the individual, fieldworker, and PSU levels comprise the independent variable. The model is defined as:


$$\begin{array}{l} \text{log}(\frac{\pi_{\text{i}\left(\text{j},\text{kl}\right)}}{\text{1-}\pi_{\text{i}\left(\text{j},\text{kl}\right)}})\text{=}\beta_{\text{0}}\text{+}\beta_{1}^{T}{\text{x}}_{\text{i}\left(\text{j},\text{kl}\right)}\text{+}\beta_{2}^{T}{\text{x}}_{\text{0k}}\text{+}\;\varepsilon_{\text{0j}}\text{+}\varepsilon_{\text{0k}}\text{+}\varepsilon_{\text{0l}} \end{array}$$

In this case, π_i(j, kl)_ denotes the likelihood that, for one of the dependent variable categories as previously described, the ith respondent questioned by the jth interviewer in the kth PSU of the lth district will get the value of 1. The coefficients of socioeconomic and demographic factors at the person level are contained in $\beta_{1}^{T}$ vector, while the coefficients of covariates at the PSU level are contained in $\beta_{2}^{T}$ vector. The error terms at the interviewer, PSU, and district levels are denoted by the symbols ε_0j_, ε_0k_, and ε_01_, in that order.


$$\begin{array}{l} \varepsilon_{\text{0j}}\sim \text{N}\left(\text{0},\sigma_{\text{0j}}^{\text{2}}\right),\varepsilon_{\text{0k}}\sim \text{N}\left(\text{0},\sigma_{\text{0k}}^{\text{2}}\right),\varepsilon_{\text{0j}}\sim \text{N}\left(\text{0},\sigma_{\text{0l}}^{\text{2}}\right)\;; \end{array}$$

where σ^2^ are variances at different levels.

**Figure 1:**
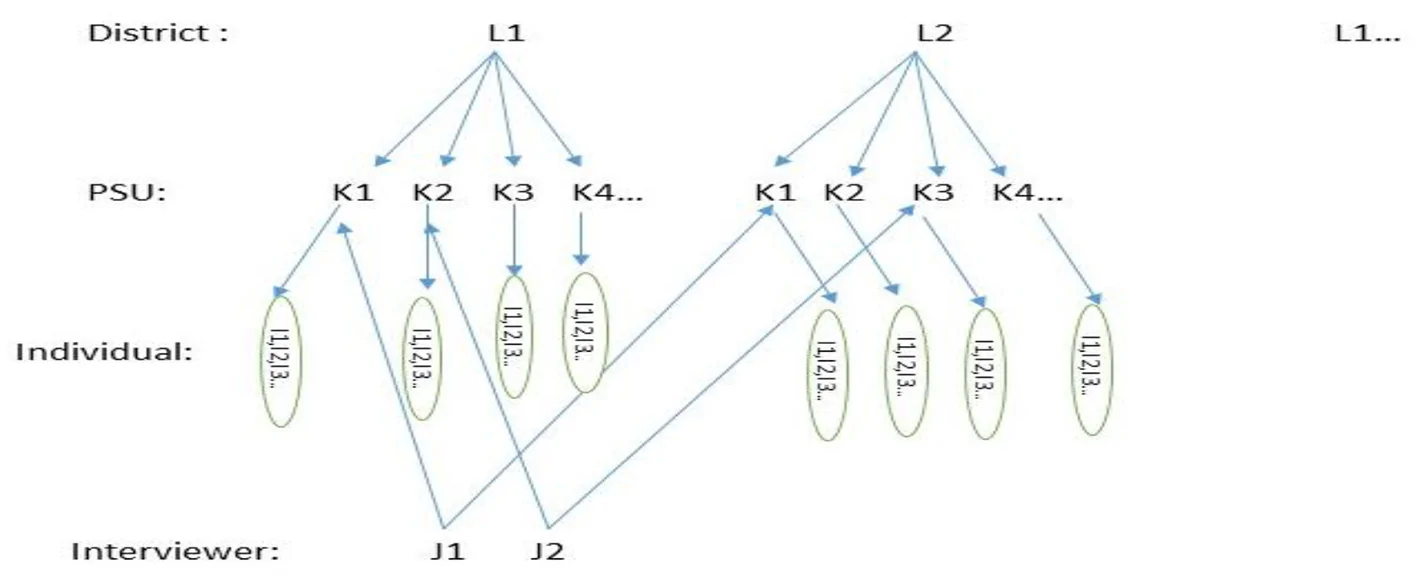
Flowchart of a hierarchical structure.

The Markov Chain Monte Carlo (MCMC) estimation process was used to estimate the cross-classified random intercept models, and the model was executed using the RunMLwiN program in STATA 16.0. Appropriate sampling weights were applied in the analysis to ensure representative estimates. In this study, ICC was calculated, which represents the share of the total variance in the outcome variable at all levels. As the study used the logit link function in the cross-classified model analysis, the individual-level variance is a fixed value, i.e., 3.29, and has been used in NFHS data (Singh et al., 2024). Figure 1 shows the flowchart of the hierarchical structure:

At the district level, ICC calculated the relationship between two distinct women living in the same district but being questioned by several field workers at various PSUs.


$$\begin{array}{l} \text{ICC particular level}\\ =\frac{selected\;level\;variance\;}{\left(District\;level\;variance+PSU\;level\;variance+Investigator\;level\;variance+individual\;level\;variance\right)}\\ \text{ICC at FW level}\\ =\frac{Fieldworker\;level\;variance\;}{\left(District\;level\;variance+PSU\;level\;variance+Investigator\;level\;variance+individual\;level\;variance\right)}\\ =\left[\frac{\sigma_{0j}^{2}}{\sigma_{0l}^{2}+\sigma_{0k}^{2}+\sigma_{0i}^{2}+3.29}\right]\\ \text{ICC at District level}\\ =\frac{District\;level\;variance\;}{\left(District\;level\;variance+PSU\;level\;variance+Investigator\;level\;variance+individual\;level\;variance\right)}\\ =\left[\frac{\sigma_{0l}^{2}}{\sigma_{0l}^{2}+\sigma_{0k}^{2}+\sigma_{0j}^{2}+3.29}\right]\\ \text{ICC at PSU level}\\ =\frac{PSU\;level\;variance\;}{\left(District\;level\;variance+PSU\;level\;variance+Investigator\;level\;variance+individual\;level\;variance\right)}\\ =\;\left[\frac{\sigma_{0k}^{2}}{\sigma_{0l}^{2}+\sigma_{0k}^{2}+\sigma_{0j}^{2}+3.29}\right] \end{array}$$

The ICC at each level represents the correlation in the outcome between two observations sharing the same higher-level unit. The interviewer-level ICC reflects similarity between observations handled by the same interviewer, the PSU-level ICC reflects similarity between observations within the same PSU but handled by different interviewers, and the district-level ICC reflects similarity between observations within the same district but belonging to different PSUs and handled by different interviewers.

## 3 Result

### 3.1 Profile of the Interviewer, NFHS-5

The data presented in Table 1 outline the demographic profile of interviewers involved in the study. Of the total 2,950 investigators, 43% hailed from urban areas. A majority of interviewers, approximately 61%, fell within the age range of 21–25 years, with an average age of 24.76 years (standard deviation = 4.42 years). The vast majority of interviewers, accounting for 87%, had never been married, and 92% had no children. A majority of interviewers (89%) are highly educated. In terms of religious affiliation, the majority of interviewers identified themselves as Hindu, comprising 73% of the total, followed by Christianity (11%). Regarding caste distribution, approximately 31% belonged to other backward classes (OBCs), 25% were from scheduled castes (SCs), and 23% were from scheduled tribes (STs). Only 22% of interviewers had previous experience working in NFHS, and 28% had participated in surveys other than NFHS in the past. Notably, around a quarter (24%) of the interviewees were currently hired by an agency at the time of their recruitment for NFHS. Approximately 28% of the interviewers are Hindi speakers.

**Table 1:** Profile of interviewers, NFHS-5 (2019–2021).

Interviewer characteristics	Frequency	Percent
Residence type		
Urban	1,273	43.15
Rural	1,677	56.85
Residence state		
Jammu and Kashmir	125	4.24
Himachal Pradesh	85	2.88
Punjab and Chandigarh	94	3.19
Uttarakhand	25	0.85
Haryana	5	0.17
NCT of Delhi	51	1.73
Rajasthan	19	0.64
Uttar Pradesh	309	10.47
Bihar	45	1.53
Sikkim	26	0.88
Arunachal Pradesh	138	4.68
Nagaland	18	0.61
Manipur	1	0.03
Mizoram	57	1.93
Tripura	3	0.1
Meghalaya	104	3.53
Assam	210	7.12
West Bengal	148	5.02
Jharkhand	9	0.31
Odisha	197	6.68
Chhattisgarh	35	1.19
Madhya Pradesh	200	6.78
Gujarat	215	7.29
Maharashtra	169	5.73
Andhra Pradesh and Andaman	107	3.63
Karnataka	149	5.05
Goa	20	0.68
Lakshadweep and Kerala	30	1.02
Tamil Nadu and Puducherry	138	4.68
Telangana	218	7.39
Age		
15–20	261	8.85
21–25	1,802	61.08
26–30	673	22.81
30+	214	7.25
Marital status		
Never married	2,569	87.08
Ever married	381	12.92
Children ever born		
No child	2,734	92.68
Having child	216	7.32
Education		
Primary	181	6.14
Secondary	135	4.58
Higher	2,634	89.29
Religion		
Hindu	2,160	73.22
Muslim	255	8.64
Christian	331	11.22
Sikh	80	2.71
Other	124	4.2
Caste		
SC	674	25.08
ST	615	22.89
OBC	858	31.93
Other	540	20.1
Ever worked on NFHS (DHS)		
Yes	651	22.07
No	2,299	77.93
Worked on any survey		
Yes	830	28.14
No	2,120	71.86
Employed with agency at time of NFHS (DHS)		
Yes	714	24.2
No	2,236	75.8
Language of the interviewer		
Assamese	187	6.34
Bengali	173	5.86
Gujarati	201	6.81
Hindi	843	28.58
Kannada	120	4.07
Kashmiri	119	4.03
Konkani	20	0.68
Malayalam	34	1.15
Manipuri	1	0.03
Marathi	166	5.63
Nepali	29	0.98
Oriya	201	6.81
Punjabi	92	3.12
Tamil	142	4.81
Telugu	299	10.14
Urdu	10	0.34
English	8	0.27
Garo	26	0.88
Khasi	71	2.41
Mizo	55	1.86
Other	153	5.19
Total no. of field investigator	**2,950**	

### 3.2 Fieldworker-respondent duo attributes

In Table 2, a comparative analysis of interviewers' characteristics concerning residence, marital status, age, religion, and caste in relation to women respondents reveals notable differences and commonalities. Specifically, 13.8% of field investigators had a different state of residence compared to respondents, while 43.9% of fieldworkers had a different type of place of residence than respondents. 12.5% of fieldworkers differed in marital status from respondents. Age-wise, 75.1% of investigators were younger than respondents. Regarding religion, 70.9% of interviewers and respondents belonged to the same religion, and only 36.7% of interviewers and respondents had the same caste. Approximately 83.3% of respondents and their interviewers spoke the same native language. About equal percentages of interviewers conducted a low, moderate, and high number of interviews, respectively. These findings provide insights into demographic dynamics between investigators and respondents.

**Table 2:** Fieldworker-respondent duo attributes, NFHS-5 (2019–2021).

Background variables	*N* = 140,549	
	Frequency	Percent
Residence (state)		
Different	19,423	13.82
Same	121,126	86.18
Residence (type)		
Different	61,706	43.90
Same	78,843	56.10
Marital status		
Different	17,548	12.49
Same	123,001	87.51
Age		
Younger	105,581	75.12
Same	6,006	4.27
Older	28,962	20.61
Religion		
Different	40,911	29.11
Same	99,638	70.89
Caste		
Different	88,997	63.32
Same	51,552	36.68
Language		
Different	23,414	16.66
Same	117,135	83.34
Number of interviews		
Low no. of interviews	47,493	33.79
Moderate no. of interviews	46,426	33.03
High no. of interviews	46,630	33.18

### 3.3 Variance at interviewer level: findings from a cross-classified four-level multilevel model

Table 3 displays the estimated district, PSU, and interviewer-level intraclass correlation coefficients for reporting opting for traditional contraceptives after no contraceptive method use, childbirth, or pregnancy termination. These outcomes were derived from a four-level random intercept multivariable logistic regression model. Following the adjustment of respondents' and interviewers' background variables, detailed estimates of each of the outcome variables and their constituent domains are provided in Table 3. The analysis has found a significant impact of the characteristics of interviewers on the reporting of opting for traditional contraceptives after women have had a birth, pregnancy termination, and non-use of contraception. The interviewer effect for the outcome variable indicates that approximately 19% of the variability in the outcome variable is attributable to differences between districts, approximately 13% of the variability in the outcome variable is due to differences between PSUs, and approximately 29% of the variability in the outcome variable is due to interviewers.

**Table 3:** Outlining the percentages of variance attribution for reporting of traditional contraceptive method use after childbirth, pregnancy termination, and no method use, at district, PSU, and interviewer levels using the cross-classified four-level model NFHS-5, 2019–2021.

Outcome	District			PSU			Interviewer		
	Variance	95% confidence interval	ICC (%)	Variance	95% confidence interval	ICC (%)	Variance	95% confidence interval	ICC (%)
Traditional contraceptive methods	1.54	1.341, 1.739	18.50	1.12	0.962, 1.279	13.46	2.37	2.220, 2.528	28.50

### 3.4 Percentage of women reporting traditional contraceptive use after birth, pregnancy termination, and no method use by interviewer characteristics

The highest reporting was observed among interviewers aged 21–25 years and those aged 30 and older. Interviewers with secondary education recorded the lowest reporting of traditional method use (19.60%) compared with other interviewers.

Reporting of traditional contraceptive use among women by the interviewer revealed notable variation across interviewer characteristics. Interviewers with secondary education recorded lower reporting of traditional contraceptive use (20%) as compared to interviewers who completed higher education and above (33%). The findings indicate limited variation in reporting by the place of residence of the interviewer and whether the interviewer had children or not.

Reporting was slightly higher among interviewers who were employed by the agency than among those who were not.

Reporting was relatively lower when the interviewer and respondent had different marital statuses (30%), whereas it was higher when both shared the same marital status (33%). When the interviewer was younger than the respondent, the reporting of traditional method use was relatively higher than that of women of the same and higher age than the interviewer. Reporting of traditional contraceptive use is significantly varied by interviewers' religion, with higher reporting when religion is the same.

### 3.5 Multilevel cross-classified adjusted model, for reporting of traditional contraceptive use after women have had a birth, pregnancy termination, and no method use

Table 4 shows the results obtained from bivariate analysis and multilevel cross-classified model for women who reported opting for traditional contraceptives following birth, pregnancy termination, and no method use, by interviewer characteristics. Interviewers aged 30 years and above showed a positive effect, with women interviewed by those aged 30 years or older being more likely to report traditional contraceptive use (adjusted odds ratio (AOR) = 1.40, *p* < 0.01), compared to the younger group (15–20 years), while no significant differences were observed for the 21–25 and 26–30 groups. The religion of the interviewer was associated with differences in reporting traditional contraceptive use. Compared to Hindu interviewers, Muslim interviewers were associated with significantly lower reporting (AOR = 0.43, *p* < 0.01). Caste background is also influenced by reporting. Women interviewed by interviewers from OBC backgrounds had a significantly lower likelihood of reporting traditional contraceptive use compared to those interviewed by non-OBC interviewers (AOR = 0.86, *p* < 0.01). Women interviewed by interviewers with secondary education were less likely to report traditional contraceptive use compared with those interviewed by primary-educated interviewers (AOR = 0.54, *p* < 0.01), while no significant difference was observed for interviewers with higher education. Previous experience in NFHS (DHS) surveys or past employment with a survey agency was not significantly associated with reporting. However, interviewers without experience of other surveys were less likely to report traditional contraceptive use (AOR = 0.90, *p* < 0.10).

**Table 4:** Percentage and multilevel cross-classified adjusted odds ratio of women reporting opting for traditional contraceptives following birth, pregnancy termination, and no method used by interviewer characteristics, India, NFHS, 2019–2020.

Interviewer's characteristics	Labels	Traditional contraceptive methods used after birth, pregnancy termination and no method use (%)	Total	Chi-square	Women opting for traditional contraceptive methods after birth, pregnancy termination and no method used	
					AOR	95% confidence interval
Residence	Urban^®^	32.00	33,543	*p* ≤ 0.01		
	Rural	32.87	53,690		1.04	0.93, 1.16
Age	15–20^®^	31.63	6,438	*p* ≤ 0.01		
	21–25	33.55	53,940		1.16	0.94, 1.43
	26–30	29.89	19,767		1.01	0.80, 1.27
	30+	32.96	7,088		1.40^***^	1.08, 1.83
Marital status	Never married^®^	31.47	11,440	*p* = 0.277		
	Ever married	32.69	75,793		1.02	0.85, 1.22
Children ever born	No child^®^	32.60	80,828	*p* = 0.980		
	Having child	31.71	6,405		0.84	0.66, 1.06
Education	Primary^®^	32.43	6331	*p* ≤ 0.01		
	Secondary	19.6	1518		0.54^***^	0.29, 0.99
	Higher	32.79	79385		0.99	0.82, 1.19
Religion	Hindu^®^	33.39	72643	*p* ≤ 0.01		
	Muslim	22.39	7700		0.43^***^	0.33, 0.56
	Other	34.81	6891		0.94	0.80, 1.12
Caste	SC^®^	32.81	23766	*p* ≤ 0.01		
	ST	33.00	8331		0.95	0.78, 1.16
	OBC	32.11	29864		0.86^***^	0.76, 0.98
	Other	36.5	17220		1.10	0.95, 1.27
Ever worked on NFHS	Yes^®^	32.39	18828	*p* = 0.023		
	No	32.57	68406		0.96	0.83, 1.12
another survey	Yes	33.52	28127	*p* ≤ 0.01		
	No	32.06	59107		0.95	0.83, 1.08
Working with agency	Yes^®^	32.97	22066	*p* ≤ 0.01		
	No	32.39	65168		1.01	0.89, 1.15
Number of interviews	Low^®^	29.28	27,743	*p* ≤ 0.01		
	Moderate	33.58	29,758		1.35^***^	1.19, 1.52
	High	34.52	29,732		1.22^**^	1.05, 1.43
Ever worked on NFHS	Yes^®^	32.39	18,828	*p* = 0.023		
No	32.57	68,406	1.03	0.89, 1.18
Worked another survey	Yes^®^	33.52	28,127	*p* ≤ 0.01		
	No	32.06	59,107		0.90^*^	0.80, 1.01
Working with agency	Yes^®^	32.97	22,066	*p* ≤ 0.01		
	No	32.39	65,168		1.00	0.88, 1.12
Residence of interviewer to respondent (state)	Different^®^	33.13	13,577	*p* ≤ 0.01		
	Same	32.42	73,657		0.82^**^	0.67, 0.98
Type of residence of interviewer to respondent (type)	Different^®^	32.4	37,829	*p* ≤ 0.01		
	Same	32.63	49,405		0.99	0.91, 1.08
Marital status of interviewer to respondent	Different^®^	29.6	12,477	*p* ≤ 0.01		
	Same	33.02	74,756		1.42^***^	1.21, 1.65
Age of interviewer to respondent	Less^®^	33.7	56,955	*p* ≤ 0.01		
	Same	31.27	4,783		0.89^**^	0.80, 0.98
	More	30.16	25,496		0.86^***^	0.80, 0.92
Religion of interviewer to respondent	Different^®^	29.46	23,367	*p* ≤ 0.01		
	Same	33.66	63,867		1.21^***^	1.11, 1.31
Caste of interviewer to respondent	Different^®^	33.01	56,304	*p* ≤ 0.01		
	Same	31.58	33,416		0.94^**^	0.88, 0.10
Language of interviewer to respondent	Different^®^	25.98	8,828	*p* ≤ 0.01		
	Same	33.27	78,406		1.05	0.99, 1.10
	Total (87,234)					

^*^p < 0.05,

^**^p < 0.01,

^***^p < 0.001.

Interviewer proficiency in administering the NFHS-5 schedule, particularly the contraceptive calendar, had a significant positive effect on reporting. Women interviewed by those who conducted a moderate number of interviews (AOR = 1.35, *p* < 0.01) or a high number of interviews (AOR = 1.26, *p* < 0.05) were more likely to report traditional contraceptive use than those interviewed by less experienced interviewers.

Interviewer–respondent similar characteristics also influenced reporting. Women were less likely to report traditional contraceptive use when the interviewer was of a similar age (AOR = 0.89, *p* < 0.05) or older (AOR = 0.86, *p* < 0.01) than the respondent. Interviewers from the same state as the respondent were associated with lower reporting (AOR = 0.82, *p* < 0.01). In contrast, women were more likely to report traditional contraceptive use when the interviewer and respondent shared the same marital status (AOR = 1.42, *p* < 0.01) or religion (AOR = 1.21, *p* < 0.01). However, sharing the same caste was associated with slightly lower reporting (AOR = 0.94, *p* < 0.05).

## 4 Discussion and conclusion

This study highlights an often overlooked issue in contraceptive data collection, that is, the substantial underreporting of traditional contraceptive methods in the NFHS-5 contraceptive calendar data. Understanding these discrepancies requires careful consideration of the role played by interviewers during data collection. The present study examined the underreporting of traditional contraceptive methods through the lens of interviewer characteristics and their influence on the reporting of traditional contraceptive use following birth, pregnancy termination, and periods of non-use.

The findings reveal that interviewer characteristics, including age, educational attainment, prior survey experience, and workload, can significantly influence the accuracy of the data collected. Furthermore, similarities and differences between interviewers and respondents in characteristics such as state of residence, marital status, age, religion, and caste were found to affect the quality and completeness of contraceptive reporting.

Interviewer education exhibited a U-shaped association with reporting patterns. Women interviewed by secondary educated interviewers were less likely to report traditional contraceptive use, whereas no significant difference was observed among those interviewed by highly educated interviewers. One possible explanation is that interviewers with primary education may establish stronger rapport with respondents, particularly in rural settings, thereby encouraging more open disclosure of culturally familiar contraceptive practices. In contrast, secondary-educated interviewers may probe differently, and it may create discomfort among respondents because of perceived educational differences. Highly educated interviewers, on the other hand, may maintain greater professional neutrality, thereby avoiding both the advantages of increased rapport and the disadvantages associated with social distance. Previous research has demonstrated that interviewer-respondent educational matching can influence survey participation and response behavior (Durrant et al., 2010). Studies have further shown that educational similarity often leads to higher cooperation rates, while respondents with lower educational attainment may provide more substantive responses when interviewed by college-educated interviewers (Yang and Yu, 2008). Together, these findings suggest that educational differences between interviewers and respondents can shape disclosure patterns in complex ways.

Interviewers aged over 30 years were associated with higher reporting of traditional contraceptive use. When interviewers and respondents belong to the same state of residence, the resulting social proximity appeared to have a negative effect on reporting. In close-knit communities, respondents may fear breaches of confidentiality, which can discourage the disclosure of sensitive information. This finding is consistent with earlier research by Anglewicz et al. (2019), who noted that many large-scale surveys deliberately recruit interviewers from outside the study community to minimize concerns about confidentiality. Nevertheless, their studies also demonstrated that interviewer-respondent familiarity can significantly influence the reporting of sensitive behaviors, including traditional contraceptive use and unwanted births. Rural respondents, who are more likely than urban respondents to know local interviewers personally, often exhibit higher levels of honest disclosure when interviewed by someone from a similar rural background (Anglewicz et al., 2019).

The study also found that interviewer-respondent similarity in marital status positively influenced the reporting of traditional contraceptive use. This finding aligns with previous evidence showing that respondents are more likely to cooperate and engage meaningfully in surveys when interviewed by individuals who share similar demographic characteristics (Webster, 1996).

With respect to religion, interviewers belonging to the Muslim religion were negatively associated with the reporting of traditional contraceptive use, suggesting that interviewer-respondent religious dynamics may influence reporting behavior. The observed variation may reflect interviewer-respondent interaction dynamics rather than inherent religious characteristics. At the same time, interviewer-respondent religious concordance was associated with higher reported, suggesting that a shared religious identity may create trust and a greater sense of comfort during the interview process.

In contrast, interviewer-respondent similarity in caste was associated with lower reporting of traditional contraceptive use. This may be attributed to concerns about social judgment or belief of a breach of confidentiality within caste-based social networks, where privacy cannot be maintained. The contrasting effects of religious and caste concordance indicate that shared identity does not uniformly promote disclosure; rather, its influence depends on the social meaning and stigma attached to specific identities and behaviors.

Although it may be assumed that matching interviewers and respondents based on demographic characteristics would improve data quality, the existing evidence provides limited support for this assumption. In some cases, deliberate matching may even introduce new sources of bias to survey responses (Davis et al., 2010). Previous research has demonstrated that interviewer-respondent acquaintance is associated with a range of family planning outcomes, highlighting the need for careful consideration of interviewer recruitment and deployment strategies in survey design (Anglewicz et al., 2019). Furthermore, studies have shown that collecting contraceptive calendar data requires substantially more time than administering conventional questionnaires. Calendar-based data collection has been reported to take approximately twice as long as traditional survey approaches (Engel et al., 2001) and, when recall aids are used, approximately 12 min longer on average than standard questionnaire-based interviews (van der Vaart, 1996).

Cultural barriers may further limit open discussion of sensitive reproductive health topics, particularly when traditional contraceptive methods are practiced privately and remain outside formal health care systems. In such settings, respondents may be reluctant to disclose their contraceptive behaviors during interviews, especially when other household members are present, thereby contributing to underreporting (Malik et al., 2023). Older interviewers may be perceived as more experienced and trustworthy, which could facilitate respondent cooperation. However, age differences between interviewers and respondents may also influence reporting behavior. Younger women, for example, may feel less comfortable discussing intimate reproductive matters with substantially older interviewers whom they perceive as moral elders. Such generational differences may reduce willingness to disclose privately practiced contraceptive methods. Previous studies have similarly reported that younger respondents may underreport sensitive behaviors when interviewed by older interviewers because of concerns about judgment or social desirability (Rozelle et al., 2023).

Research has also shown that older respondents often experience greater difficulty understanding survey questions than younger respondents. Interestingly, this challenge becomes substantially greater when the interviewer is considerably younger, increasing by approximately 3-fold compared with interviews conducted by older interviewers (Życzyńska-Ciołek and Kołczyńska, 2021). The lower reporting of traditional contraceptive methods among respondents interviewed by Muslim interviewers may also reflect broader religious norms and beliefs that discourage contraceptive use in certain contexts (Alomair et al., 2023; Shumayla and Kapoor, 2017). Previous studies have highlighted similar interviewer effects. For example, in Egypt, Muslim women reported higher levels of religiosity when interviewed by veiled women (Blaydes and Gillum, 2013). Similarly, in Morocco, religious respondents tended to provide more pious responses to interviewers wearing a hijab and less religious responses to interviewers with a more neutral appearance. These findings suggest that respondents may modify their responses to align with perceived expectations, social norms, and cultural dynamics associated with an interviewer's religious identity or appearance (Benstead, 2014).

Interviewer experience is another important determinant of data quality. Experienced interviewers are generally better equipped to manage complex and sensitive topics and are therefore more likely to collect consistent and reliable data. Interviewer effects are particularly pronounced in reproductive health surveys that require detailed recall, such as questions regarding contraceptive use and duration (Elksabi and Khan, 2022). Research shows that interviewers usually become faster at conducting interviews as they gain more experience. Compared to their first interview, later interviews often take less time because interviewers become more familiar with questionnaire and survey procedures. However, being faster may also mean that interviewers ask questions more quickly, spend less time explaining questions, or do less probing for additional information. As a result, respondents may provide less detailed answers, which could affect the quality and accuracy of the data collected (Olson and Peytchev, 2007). These findings underscore that the underreporting of traditional contraceptive use is not solely a consequence of respondent recall limitations or reluctance to disclose information. Rather, it is also shaped by the extent to which interviewers are able to establish rapport, foster trust, and effectively probe for complete responses. The results highlight the importance of considering both interviewer and respondent characteristics when designing and implementing large-scale surveys.

From a policy perspective, these findings suggest that the NFHS-5 contraceptive calendar may underreport the usage of traditional contraceptive methods, potentially leading to inaccurate assessments of family planning behaviors and needs. Such underreporting may distort estimates of contraceptive prevalence, unmet need for family planning, method satisfaction, and contraceptive continuation rates, thereby affecting program planning and service delivery. To improve data quality, interviewer recruitment and deployment strategies should take into account contextual and demographic factors that may influence respondent disclosure. In addition, interviewer training may benefit from being extended beyond technical instruction on contraceptive calendar administration to include communication skills and rapport-building techniques that may facilitate respondent disclosure. However, the effectiveness of such training requires further empirical evaluation.

Despite its contributions, this study has several limitations. Although multilevel cross-classified models were used to account for clustering at the interviewer level, unmeasured factors such as respondents' attitudes and the degree of privacy during interviews may still have influenced the findings. Furthermore, the contraceptive calendar relies on retrospective self-reported information and is therefore susceptible to recall bias. It is also possible that some respondents do not perceive traditional methods as legitimate contraceptive practices and therefore fail to report them, even when interviewers make efforts to obtain complete information.

Future research should further investigate how underreporting influences our understanding of contraceptive dynamics over time and the implications for demographic and public health research. Particular attention should be given to the role of interviewer-respondent characteristics in shaping the reporting of sensitive and frequently underreported reproductive health behaviors and outcomes.

## Data Availability

Publicly available datasets were analyzed in this study. This data can be found here: https://dhsprogram.com/data/available-datasets.cfm.

## Footnotes

1 https://www.nfhsiips.in/nfhsuser/index.php (Accessed February 5, 2026).

## References

[B1] Alomair N. Alageel S. Davies N. Bailey J. V. (2023). Muslim women's views and experiences of family planning in Saudi Arabia: a qualitative study. BMC Women's Health 23:625. doi: 10.1186/s12905-023-02786-2 38007464 PMC10675866

[B2] Anglewicz P. Akilimali P. Eitmann L. P. Hernandez J. Kayembe P. (2019). The relationship between interviewer-respondent familiarity and family planning outcomes in the Democratic Republic of Congo: a repeat cross-sectional analysis. BMJ Open 9:e023069. doi: 10.1136/bmjopen-2018-023069 30670510 PMC6348299

[B3] Benstead L. J. (2014). Does interviewer religious dress affect survey responses? Evidence from Morocco. Polit. Relig. 7, 734–760. doi: 10.1017/S1755048314000455

[B4] Bertrand J. T. Ross J. Glover A. L. (2022). Declining yet persistent use of traditional contraceptive methods in low- and middle-income countries. J. Biosoc. Sci. 54, 742–759. doi: 10.1017/S0021932021000341 34269170 PMC11753471

[B5] Blaydes L. Gillum R. M. (2013). Religiosity-of-interviewer effects: assessing the impact of veiled enumerators on survey response in Egypt. Polit. Relig. 6, 459–482. doi: 10.1017/S1755048312000557

[B6] Callahan R. L. Becker S. (2012). The reliability of calendar data for reporting contraceptive use: evidence from Rural Bangladesh. Stud. Fam. Plan. 43, 213–222. doi: 10.1111/j.1728-4465.2012.00319.x 23185864 PMC3628694

[B7] Cannell C. F. Fowler F. J. Marquis K. H. (1968). The Influence of Interviewer and Respondent Psychological and Behavioral Variables on the Reporting in Household Interviews. Washington, DC: U.S. Government Printing Office. 10.1037/e416812004-001 5301315

[B8] Davis R. E. Couper M. P. Janz N. K. Caldwell C. H. Resnicow K. (2010). Interviewer effects in public health surveys. Health Educ. Res. 25, 14–26. doi: 10.1093/her/cyp046 19762354 PMC2805402

[B9] Durrant G. B. Groves R. M. Staetsky L. Steele F. (2010). Effects of interviewer attitudes and behaviors on refusal in household surveys. Public Opin. Q. 74, 1–36. doi: 10.1093/poq/nfp098

[B10] Elkasabi M. Assaf S. (2021). Accumulating Contraceptive Calendars Across Surveys. Rockville, Maryland: ICF.

[B11] Elksabi M. Khan A. (2022). Modeling Interviewer Effects in DHS Surveys. Rockville, Maryland: ICF.

[B12] Engel L. S. Keifer M. C. Zahm S. H. (2001). Comparison of a traditional questionnaire with an icon/calendar-based questionnaire to assess occupational history. Am. J. Indus. Med. 40, 502–511. doi: 10.1002/ajim.1118 11675619

[B13] Glasner T. Van Der Vaart W. (2009). Applications of calendar instruments in social surveys: a review. Qual. Quant. 43, 333–349. doi: 10.1007/s11135-007-9129-8 20046840 PMC2798968

[B14] Groves R. M. (1989). Survey Errors and Survey Costs, 1st Edn. New York, NY: John Wiley & Sons.

[B15] Kühne S. (2023). Interpersonal perceptions and interviewer effects in face-to-face surveys. Sociol. Methods Res. 52, 299–334. doi: 10.1177/0049124120926215

[B16] Kumar K. Singh A. Tsui A. (2022). Measuring contraceptive use in India: implications of recent fieldwork design and implementation of the National Family Health Survey. DemRes 47, 73–110. doi: 10.4054/DemRes.2022.47.4

[B17] Mairiga A. G. Garba A. Kullima A. Bako B. (2012). The practice of traditional family planning among rural Kanuri communities of Northeastern Nigeria. Int. J. Biol. Med. Res. 3, 1277–1280.

[B18] Malik M. Girotra S. Zode M. Basu S. (2023). Patterns and predictors of abortion care-seeking practices in India: evidence from a Nationally Representative Cross-Sectional Survey (2019-2021). Cureus 15:e41263. doi: 10.7759/cureus.41263 37529821 PMC10390032

[B19] Marston C. Renedo A. Nyaaba G. N. Machiyama K. Tapsoba P. Cleland J. . (2017). Improving the measurement of fertility regulation practices: findings from qualitative research in Ghana. Int. Perspect. Sex. Reprod. Health 43, 111–119. doi: 10.1363/43e4517 29553472

[B20] Namasivayam V. Dehury B. Prakash R. Becker M. Anand P. Mishra A. . (2023). Understanding the rise in traditional contraceptive methods use in Uttar Pradesh, India. Reprod. Health 20:8. doi: 10.1186/s12978-022-01547-y 36609308 PMC9817250

[B21] National Family Health Survey (2022). NFHS-5 National report. Mumbai: IIPS.

[B22] Olson K. Peytchev A. (2007). Effect of interviewer experience on interview pace and interviewer attitudes. Public Opin. Q. 71, 273–286. doi: 10.1093/poq/nfm007

[B23] Polis C. B. Bradley S. E. K. Bankole A. Onda T. Croft T. Singh S. . (2016). Contraceptive Failure Rates in the Developing World: An Analysis of Demographic and Health Survey Data in 43 Countries. New York, NY: Guttmacher Institute. Available online at: https://www.guttmacher.org/report/contraceptive-failure-rates-in-developing-world (Accessed August 30, 2025).

[B24] Prakash R. Mahapatra T. Singh S. Mishra A. K. (2022). Increasing-traditional-method-users-in-UP-and-Bihar. New Delhi: FP-MLE Consortium.

[B25] Rabiu A. Rufa'i A. A. (2018). The role of traditional contraceptive methods in family planning among women attending primary health care centers in Kano. Ann. Afr. Med. 17:189. doi: 10.4103/aam.aam_60_17 30588932 PMC6330776

[B26] Ram F. Shekhar C. Chowdhury B. (2014). Use of traditional contraceptive methods in India & its socio-demographic determinants. Indian J Med Res. 140, S17–S28. doi: 10.4103/0971-5916.151034 25673538 PMC4345748

[B27] Rossier C. Senderowicz L. Soura A. (2014). Do natural methods count? Underreporting of natural contraception in Urban Burkina Faso. Stud. Fam. Plan. 45, 171–182. doi: 10.1111/j.1728-4465.2014.00383.x 24931074

[B28] Rozelle J. W. Meyer M. J. McKenna A. H. Obaje H. Kraemer J. D. (2023). The effect of interviewer-respondent age difference on the reporting of sexual activity in the Demographic and Health Surveys: analysis of data from 21 countries. J. Glob. Health 13:04002. doi: 10.7189/jogh.13.04002 36651233 PMC9850865

[B29] Shumayla S. Kapoor S. (2017). Knowledge, attitude, and practice of family planning among Muslim women of North India. Int. J. Med. Sci. Public Health 6:1. doi: 10.5455/ijmsph.2017.1266308122016

[B30] Singh S. Dwivedi L. K. Jana S. (2024). Fieldworker effects on quality of data collected on sensitive questions in National Family Health Surveys: an analysis based on intimate partner violence in India. SSM Popul. Health 25:101557. doi: 10.1016/j.ssmph.2023.101557 38089851 PMC10711512

[B31] United Nations Population Division (2024). Population Division Data Portal. Available online at: https://population.un.org/dataportal/home?df=e4c0ea9e-4d63-4913-815d-4e9524148bdc (Accessed August 25, 2025).

[B32] United Nations Department of Economic and Social Affairs, Population Division. (2024). World Contraceptive Use 2024. Available online at: https://www.un.org/development/desa/pd/data/world-contraceptive-use (Accessed August 25, 2025).

[B33] van der Vaart W. (1996). Inquiring into the past. Data quality of responses to retrospective questions. [PhD Thesis]. Research and graduation internal. Vrije Universiteit Amsterdam, Amsterdam, Netherlands.

[B34] Webster C. (1996). Hispanic and Anglo interviewer and respondent ethnicity and gender: the impact on survey response quality. J. Mark. Res. 33, 62–72. doi: 10.1177/002224379603300106

[B35] Yang M-. L. Yu R-. R. (2008). The interviewer effect when there is an education gap with the respondent: Evidence from a survey on biotechnology in Taiwan. Soc. Sci. Res. 37, 1321–1331. doi: 10.1016/j.ssresearch.2008.05.008

[B36] Zaccarin S. Rivellini G. (2002). Multilevel analysis in social research: an application of a cross-classified model. Stat. Methods Appl. 11, 95–108. doi: 10.1007/BF02511448

[B37] Życzyńska-Ciołek D. Kołczyńska M. (2021). Does interviewers' age affect their assessment of respondents' understanding of survey questions? Evidence from the European social survey. Int. J. Public Opin. Res. 33, 460–476. doi: 10.1093/ijpor/edaa015

